# Supplementary material for: Barriers and facilitators to the implementation of mental health and psychosocial support programmes following natural disasters in developing countries: A systematic review
Source: Glob Ment Health (Camb). 2023 Dec 29;11:e5. doi: 10.1017/gmh.2023.91 (PMC10808980; doi:10.1017/gmh.2023.91)

Supplementary Material

*Appendix 1: search concepts*

| Search Term | Synonym | Search Strategy |
| --- | --- | --- |
| Natural Disasters | Extreme weather events  Tsunami  Earthquake  Drought  Storm  Flood  Cyclone  Landslide  Volcanic eruption  Wildfire  Avalanche  Typhoon  Hurricane  Tidal wave  Famine  Emergencies  Relief work  Rescue work | Disaster* OR Tsunami* OR earthquake* OR drought* OR storm* OR flood* OR cyclone* OR landslide* OR volcan* OR wildfire* OR Avalanche* OR Typhoon* OR Hurricane* OR Tidal wave* OR famine* OR natural disaster* OR natural hazard* OR extreme weather OR emergenc* OR relief work OR rescue work |
| Disaster victim | Refugee  Asylum seeker  Survivor  Evacuee  Internally displaced person  IDP | Refugee* OR Asylum Seeker* OR Survivor* OR evacuee* OR displace* OR IDP |
| Mental Health | Mental disorder  Mental illness  Psychological/psychosocial wellbeing  Stress | Mental health OR mental disorder OR mental illness OR psychological OR psychosocial OR wellbeing OR well being OR stress |
| Depression | Low mood  Mood disorder  Depressive symptoms  Negative mood  Social withdrawal | Low mood OR mood disorder OR depress* OR negative mood OR social withdrawal |
| Anxiety | Anxious  Post-traumatic stress disorder/PTSD | Anx* OR post-traumatic stress OR PTSD |
| Programme | Intervention  Plan  Action  Service  Response  Mental health and psychosocial support (MHPSS)  Emotional support  Therapy  Health system  Primary care  Psychotherapy  Mental health services  Social work  Health communication  Safe space  Family support  Psychoeducation  Cognitive behavioural therapy  CBT | Program* OR intervention* OR plan* OR action* OR service* OR response* OR MHPSS OR support OR therap* OR health system* OR primary care OR primary health care OR primary healthcare OR psychotherapy OR mental health service* OR mental health care OR social work OR health communication* OR safe space* OR family support OR psychoeducation OR CBT |
| Developing countries | Low and middle income countries/ LMICs/ LICs/ MICs  Global south  Developing and emerging economies  Transitional countries  Less developed  Under developed  Small island developing states (SIDS)  Developing nations  Third world  Lami countries  Transitional countries  Names of LMICs according to the World Bank  Africa  Asia  Caribbean  West Indies  South America  Latin America  Central America  Sub-Saharan | Developing OR low* income OR middle* income OR LMIC OR LIC OR MIC OR global south OR emerging OR transition* OR SIDS OR “less* developed” OR underdeveloped OR “under* developed” OR “third world” OR “lami countr*” OR “transitional countr*” OR Afghanistan OR Albania OR Algeria OR American Samoa OR Angola OR Argentina OR Argentine Republic OR Armenia OR Azerbaijan OR Bangladesh OR Belarus OR Byelarus OR Byelorussia OR Belorussia OR Belize OR British Honduras OR Benin OR Dahomey OR Bhutan OR Bolivia OR Bosnia and Herzegovina OR Botswana Bechuanaland OR Kalahari OR Brazil OR Bulgaria OR Burkina Faso OR Burkina Fasso OR Upper Volta OR Burundi OR Ruanda-Urundi OR Cabo Verde OR Cambodia OR Cameroon OR Central African Republic OR Ubangi-Shari OR Chad OR China OR Colombia OR Comoros OR Democratic republic of Congo OR Republic of Congo OR Congo* OR Costa Rica OR Cote D’Ivoire OR Cuba OR Djibouti OR Dominica OR Dominican Republic OR Ecuador OR Egypt OR El Salvador OR Equatorial Guinea OR Spanish Guinea OR Eritrea OR Eswatini OR Ethiopia OR Abyssinia OR Fiji OR Gabon OR Gabonese Republic OR Gambia OR Georgia not “Georgia (u.s.)” OR Ghana OR Grenada OR Guatemala OR Guinea not (New Guinea or Guinea Pig* or Guinea Fowl or Guinea-Bissau or Portuguese Guinea or Equatorial Guinea) OR Guinea-Bissau OR Guyana OR British Guiana OR Haiti OR Hayti OR Honduras OR India OR Indonesia OR Dutch East Indies OR Iran OR Persia OR Iraq OR Mesopotamia OR OR Jamaica OR Jordan OR Kazakh* OR Kenya OR Kiribati OR Gilbert Islands OR Phoenix Islands OR Line Islands OR Korea OR Kosovo OR Kyrgyz Republic OR Lao* OR Lebanon OR Lebanese Republic OR Lesotho OR Liberia OR Libya OR Libyan Arab Jamahiriya OR Macedonia OR Madagascar OR Malagasy Republic OR Malawi OR Nyasaland OR Malaysia OR Maldives OR Mali OR Marshall Islands OR Mauritania OR Mauritius OR Agalega Islands OR Mexico OR United Mexican States OR Micronesia OR Moldova OR Mongolia OR Montenegro OR Morocco OR Mozambique OR Mocambique OR Portuguese East Africa OR Myanmar OR Burma OR Namibia OR German South West Africa OR Nepal OR Nicaragua OR Niger not (Aspergillus or Peptococcus or Schizothorax or Cruciferae or Gobius or Lasius or Agelastes or Melanosuchus or radish or Parastromateus or Orius or Apergillus or Parastromateus or Stomoxys) OR Nigeria OR North Macedonia OR Pakistan OR Panama OR Papua New Guinea OR German New Guinea OR British New Guinea OR Territory of Papua OR Paraguay OR Peru OR Philippines OR Philippine Islands OR Romania OR Russia OR Russian Federation OR USSR OR Union of Soviet Socialist Republics OR Soviet Union OR Rwanda OR Ruanda OR Samoa OR Sao Tome and Principe OR Senegal OR Serbia OR Sierra Leone OR Salone OR Solomon Islands OR Somalia OR South Africa OR Cape Colony OR British Bechuanaland OR Boer Republics OR Zululand OR Transvaal OR Natalia Republic OR Orange Free State OR South Sudan OR Sri Lanka OR Ceylon OR St Lucia OR Saint Lucia OR Iyonala OR Hewanora OR St Vincent and the Grenadines OR Saint Vincent and the Grenadines OR Saint Vincent or St Vincent OR Greandines OR Sudan or Suriname OR OR Dutch Guiana OR Syria* OR Tajikistan OR Tanzania OR Tanganyika OR Zanzibar OR Thailand OR Timor-Leste OR Togo OR Togolese Republic OR Togoland OR Tonga OR Tunisia OR Turkey not “Turkey (bird)” OR Anatolia OR Asia Minor OR Turkmenistan OR Tuvalu OR Uganda OR Ukraine OR Uzbekistan OR Vanuatu OR Vietnam OR West Bank and Gaza OR Yemen OR Zambia OR Zimbabwe OR Africa  OR “Sub* Saharan” OR Asia OR Caribbean OR “West Indies” OR “South America” OR “Latin America” OR “Central America” |

*Appendix 2: search strategy*

| 1. | Developing Countries/ |
| --- | --- |
| 2. | ((developing or less* developed or under developed or underdeveloped or middle income or low* income) adj (economy or economies)).ti,ab. |
| 3. | ((developing or less* developed or under developed or underdeveloped or middle income or low* income or underserved or under served or deprived or poor*) adj (countr* or nation? or population? or world)).ti,ab. |
| 4. | (low* adj (gdp or gnp or gross domestic or gross national)).ti,ab. |
| 5. | (low adj3 middle adj3 countr*).ti,ab. |
| 6. | (lmic or lmics or third world or lami countr*).ti,ab. |
| 7. | transitional countr*.ti,ab. |
| 8. | global south.ti,ab. |
| 9. | "africa south of the sahara"/ |
| 10. | africa, central/ |
| 11. | africa, eastern/ |
| 12. | africa, southern/ |
| 13. | africa, western/ |
| 14. | ("Africa South of the Sahara" or sub-Saharan Africa or subSaharan Africa).ti,ab. |
| 15. | Central Africa.ti,ab. |
| 16. | Eastern Africa.ti,ab. |
| 17. | Southern Africa.ti,ab. |
| 18. | Western Africa.ti,ab. |
| 19. | "Democratic People's Republic of Korea"/ |
| 20. | (North Korea or (Democratic People* Republic adj2 Korea)).ti,ab. |
| 21. | Cambodia/ |
| 22. | Cambodia.ti,ab. |
| 23. | Indonesia/ |
| 24. | (Indonesia or Dutch East Indies).ti,ab. |
| 25. | (Kiribati or Gilbert Islands or Phoenix Islands or Line Islands).ti,ab. |
| 26. | Laos/ |
| 27. | (Laos or (Lao adj1 Democratic Republic)).ti,ab. |
| 28. | Micronesia/ |
| 29. | Micronesia.ti,ab. |
| 30. | Mongolia/ |
| 31. | Mongolia.ti,ab. |
| 32. | Myanmar/ |
| 33. | (Myanmar or Burma).ti,ab. |
| 34. | Papua New Guinea/ |
| 35. | (Papua New Guinea or German New Guinea or British New Guinea or Territory of Papua).ti,ab. |
| 36. | Philippines/ |
| 37. | (Philippines or Philippine Islands).ti,ab. |
| 38. | "Independent State of Samoa"/ |
| 39. | ((Samoa not American Samoa) or Western Samoa or Navigator Islands or Samoan Islands).ti,ab. |
| 40. | Solomon Islands.ti,ab. |
| 41. | Timor-Leste/ |
| 42. | (Timor-Leste or East Timor or Portuguese Timor).ti,ab. |
| 43. | Vanuatu/ |
| 44. | (Vanuatu or New Hebrides).ti,ab. |
| 45. | Vietnam/ |
| 46. | (Viet Nam or Vietnam or French Indochina).ti,ab. |
| 47. | American Samoa/ |
| 48. | American Samoa.ti,ab. |
| 49. | exp China/ |
| 50. | China.ti,ab. |
| 51. | Fiji/ |
| 52. | Fiji.ti,ab. |
| 53. | Malaysia/ |
| 54. | (Malaysia or Malayan Union or Malaya).ti,ab. |
| 55. | Marshall Islands.ti,ab. |
| 56. | Nauru.ti,ab. |
| 57. | Thailand/ |
| 58. | (Thailand or Siam).ti,ab. |
| 59. | Tonga/ |
| 60. | Tonga.ti,ab. |
| 61. | (Tuvalu or Ellice Islands).ti,ab. |
| 62. | Kyrgyzstan/ |
| 63. | (Kyrgyzstan or Kyrgyz Republic or Kirghizia or Kirghiz).ti,ab. |
| 64. | Tajikistan/ |
| 65. | Tajikistan.ti,ab. |
| 66. | Ukraine/ |
| 67. | Ukraine.ti,ab. |
| 68. | Uzbekistan/ |
| 69. | Uzbekistan.ti,ab. |
| 70. | Albania/ |
| 71. | Albania.ti,ab. |
| 72. | Armenia/ |
| 73. | Armenia.ti,ab. |
| 74. | Azerbaijan/ |
| 75. | Azerbaijan.ti,ab. |
| 76. | "Republic of Belarus"/ |
| 77. | (Belarus or Byelarus or Byelorussia or Belorussia).ti,ab. |
| 78. | Bosnia-Herzegovina/ |
| 79. | (Bosnia or Herzegovina).ti,ab. |
| 80. | Bulgaria/ |
| 81. | Bulgaria.ti,ab. |
| 82. | "Georgia (Republic)"/ |
| 83. | Georgia.ti,ab. not Georgia/ |
| 84. | Kazakhstan/ |
| 85. | (Kazakhstan or Kazakh).ti,ab. |
| 86. | Kosovo/ |
| 87. | Kosovo.ti,ab. |
| 88. | Moldova/ |
| 89. | Moldova.ti,ab. |
| 90. | Montenegro/ |
| 91. | Montenegro.ti,ab. |
| 92. | "Republic of North Macedonia"/ |
| 93. | North Macedonia.ti,ab. |
| 94. | Romania/ |
| 95. | Romania.ti,ab. |
| 96. | exp Russia/ |
| 97. | "Russia (Pre-1917)"/ |
| 98. | USSR/ |
| 99. | (Russia or Russian Federation or USSR or Union of Soviet Socialist Republics or Soviet Union).ti,ab. |
| 100. | Serbia/ |
| 101. | Serbia.ti,ab. |
| 102. | Turkey/ |
| 103. | (Turkey.ti,ab. not animal/) or (Anatolia or Asia Minor).ti,ab. |
| 104. | Turkmenistan/ |
| 105. | Turkmenistan.ti,ab. |
| 106. | Belize/ |
| 107. | (Belize or British Honduras).ti,ab. |
| 108. | Bolivia/ |
| 109. | Bolivia.ti,ab. |
| 110. | El Salvador/ |
| 111. | El Salvador.ti,ab. |
| 112. | Haiti/ |
| 113. | (Haiti or Hayti).ti,ab. |
| 114. | Honduras/ |
| 115. | Honduras.ti,ab. |
| 116. | Nicaragua/ |
| 117. | Nicaragua.ti,ab. |
| 118. | Argentina/ |
| 119. | (Argentina or Argentine Republic).ti,ab. |
| 120. | Brazil/ |
| 121. | Brazil.ti,ab. |
| 122. | Colombia/ |
| 123. | Colombia.ti,ab. |
| 124. | Costa Rica/ |
| 125. | Costa Rica.ti,ab. |
| 126. | Cuba/ |
| 127. | Cuba.ti,ab. |
| 128. | Dominica/ |
| 129. | Dominica.ti,ab. |
| 130. | Dominican Republic/ |
| 131. | Dominican Republic.ti,ab. |
| 132. | Ecuador/ |
| 133. | Ecuador.ti,ab. |
| 134. | Grenada/ |
| 135. | Grenada.ti,ab. |
| 136. | Guatemala/ |
| 137. | Guatemala.ti,ab. |
| 138. | Guyana/ |
| 139. | (Guyana or British Guiana).ti,ab. |
| 140. | Jamaica/ |
| 141. | Jamaica.ti,ab. |
| 142. | Mexico/ |
| 143. | (Mexico or United Mexican States).ti,ab. |
| 144. | Panama/ |
| 145. | Panama.ti,ab. |
| 146. | Paraguay/ |
| 147. | Paraguay.mp. |
| 148. | Peru/ |
| 149. | Peru.ti,ab. |
| 150. | Saint Lucia/ |
| 151. | (St Lucia or Saint Lucia or Iyonala or Hewanorra).ti,ab. |
| 152. | "Saint Vincent and the Grenadines"/ |
| 153. | (Saint Vincent or St Vincent or Grenadines).ti,ab. |
| 154. | Suriname/ |
| 155. | (Suriname or Dutch Guiana).ti,ab. |
| 156. | Venezuela/ |
| 157. | Venezuela.ti,ab. |
| 158. | Algeria/ |
| 159. | Algeria.ti,ab. |
| 160. | Djibouti/ |
| 161. | (Djibouti or French Somaliland).ti,ab. |
| 162. | Egypt/ |
| 163. | Egypt.ti,ab. |
| 164. | Iran/ |
| 165. | (Iran or Persia).ti,ab. |
| 166. | Morocco/ |
| 167. | Morocco.ti,ab. |
| 168. | Tunisia/ |
| 169. | Tunisia.mp. |
| 170. | (Gaza or West Bank or Palestine).ti,ab. |
| 171. | Iraq/ |
| 172. | (Iraq or Mesopotamia).ti,ab. |
| 173. | Jordan/ |
| 174. | Jordan.ti,ab. |
| 175. | Lebanon/ |
| 176. | (Lebanon or Lebanese Republic).ti,ab. |
| 177. | Libya/ |
| 178. | Libya.ti,ab. |
| 179. | Afghanistan/ |
| 180. | Afghanistan.ti,ab. |
| 181. | Bangladesh/ |
| 182. | Bangladesh.ti,ab. |
| 183. | Bhutan/ |
| 184. | Bhutan.ti,ab. |
| 185. | exp India/ |
| 186. | India.ti,ab. |
| 187. | Nepal/ |
| 188. | Nepal.ti,ab. |
| 189. | Pakistan/ |
| 190. | Pakistan.ti,ab. |
| 191. | Sri Lanka/ |
| 192. | (Sri Lanka or Ceylon).ti,ab. |
| 193. | Maldives.ti,ab. [UPPER MIDDLE INCOME COUNTRIES IN SOUTH ASIA] |
| 194. | Angola/ |
| 195. | Angola.ti,ab. |
| 196. | Benin/ |
| 197. | Benin.ti,ab. |
| 198. | Cameroon/ |
| 199. | (Cameroon or Kamerun or Cameroun).ti,ab. |
| 200. | Cape Verde/ |
| 201. | (Cape Verde or Cabo Verde).ti,ab. |
| 202. | Comoros/ |
| 203. | (Comoros or Glorioso Islands or Mayotte).ti,ab. |
| 204. | Congo/ |
| 205. | (Congo not ((Democratic Republic adj3 Congo) or congo red or crimean-congo)).ti,ab. |
| 206. | Cote d'Ivoire/ |
| 207. | (Cote d'Ivoire or Cote dIvoire or Ivory Coast).ti,ab. |
| 208. | Eswatini/ |
| 209. | (eSwatini or Swaziland).ti,ab. |
| 210. | Ghana/ |
| 211. | (Ghana or Gold Coast).ti,ab. |
| 212. | Kenya/ |
| 213. | (Kenya or East Africa Protectorate).ti,ab. |
| 214. | Lesotho/ |
| 215. | (Lesotho or Basutoland).ti,ab. |
| 216. | Mauritania/ |
| 217. | Mauritania.ti,ab. |
| 218. | Nigeria/ |
| 219. | Nigeria.ti,ab. |
| 220. | (Sao Tome adj2 Principe).ti,ab. |
| 221. | Senegal/ |
| 222. | Senegal.ti,ab. |
| 223. | Tanzania/ |
| 224. | (Tanzania or Tanganyika or Zanzibar).ti,ab. |
| 225. | Zambia/ |
| 226. | (Zambia or Northern Rhodesia).ti,ab. |
| 227. | Zimbabwe/ |
| 228. | (Zimbabwe or Southern Rhodesia).ti,ab. |
| 229. | Botswana/ |
| 230. | (Botswana or Bechuanaland or Kalahari).ti,ab. |
| 231. | Equatorial Guinea/ |
| 232. | (Equatorial Guinea or Spanish Guinea).ti,ab. |
| 233. | Gabon/ |
| 234. | (Gabon or Gabonese Republic).ti,ab. |
| 235. | Mauritius/ |
| 236. | (Mauritius or Agalega Islands).ti,ab. |
| 237. | Namibia/ |
| 238. | (Namibia or German South West Africa).ti,ab. |
| 239. | South Africa/ |
| 240. | (South Africa or Cape Colony or British Bechuanaland or Boer Republics or Zululand or Transvaal or Natalia Republic or Orange Free State).ti,ab. |
| 241. | Syria/ |
| 242. | (Syria or Syrian Arab Republic).ti,ab. |
| 243. | Yemen/ |
| 244. | Yemen.ti,ab. |
| 245. | Burkina Faso/ |
| 246. | (Burkina Faso or Burkina Fasso or Upper Volta).ti,ab. |
| 247. | Burundi/ |
| 248. | (Burundi or Ruanda-Urundi).ti,ab. |
| 249. | Central African Republic/ |
| 250. | (Central African Republic or Ubangi-Shari).ti,ab. |
| 251. | Chad/ |
| 252. | Chad.ti,ab. |
| 253. | "Democratic Republic of the Congo"/ |
| 254. | (((Democratic Republic or DR) adj2 Congo) or Congo-Kinshasa or Belgian Congo or Zaire or Congo Free State).ti,ab. |
| 255. | Eritrea/ |
| 256. | Eritrea.ti,ab. |
| 257. | Ethiopia/ |
| 258. | (Ethiopia or Abyssinia).ti,ab. |
| 259. | Gambia/ |
| 260. | Gambia.ti,ab. |
| 261. | Guinea/ |
| 262. | (Guinea not (New Guinea or Guinea Pig* or Guinea Fowl or Guinea-Bissau or Portuguese Guinea or Equatorial Guinea)).ti,ab. |
| 263. | Guinea-Bissau/ |
| 264. | (Guinea-Bissau or Portuguese Guinea).ti,ab. |
| 265. | Liberia/ |
| 266. | Liberia.ti,ab. |
| 267. | Madagascar/ |
| 268. | (Madagascar or Malagasy Republic).ti,ab. |
| 269. | Malawi/ |
| 270. | (Malawi or Nyasaland).ti,ab. |
| 271. | Mali/ |
| 272. | Mali.ti,ab. |
| 273. | Mozambique/ |
| 274. | (Mozambique or Mocambique or Portuguese East Africa).ti,ab. |
| 275. | Niger/ |
| 276. | (Niger not (Aspergillus or Peptococcus or Schizothorax or Cruciferae or Gobius or Lasius or Agelastes or Melanosuchus or radish or Parastromateus or Orius or Apergillus or Parastromateus or Stomoxys)).ti,ab. |
| 277. | Rwanda/ |
| 278. | (Rwanda or Ruanda).ti,ab. |
| 279. | Sierra Leone/ |
| 280. | (Sierra Leone or Salone).ti,ab. |
| 281. | Somalia/ |
| 282. | (Somalia or Somaliland).ti,ab. |
| 283. | South Sudan/ |
| 284. | South Sudan.ti,ab. |
| 285. | Sudan/ |
| 286. | Sudan.ti,ab. |
| 287. | Togo/ |
| 288. | (Togo or Togolese Republic or Togoland).ti,ab. |
| 289. | Uganda/ |
| 290. | Uganda.ti,ab. |
| 291. | or/1-290 [ALL LMICs] |
| 292. | (Disaster* or Tsunami* or earthquake* or drought* or storm* or flood* or cyclone* or landslide* or volcan* or wildfire* or Avalanche* or Typhoon* or Hurricane* or Tidal wave* or famine* or natural disaster* or natural hazard* or extreme weather or emergenc* or relief work or rescue work).mp. [mp=ti, ab, hw, tc, id, ot, tm, mf, tn, dm, dv, kf, fx, dq, cw, nm, ox, px, rx, an, ui, sy] |
| 293. | (Mental health or mental disorder or mental illness or psychological or psychosocial or wellbeing or well being or stress).mp. [mp=ti, ab, hw, tc, id, ot, tm, mf, tn, dm, dv, kf, fx, dq, cw, nm, ox, px, rx, an, ui, sy] |
| 294. | (Program* or intervention* or plan* or action* or service* or response* or MHPSS or support or therap* or health system* or primary care or primary health care or primary healthcare or psychotherapy or mental health service* or mental health care or social work or health communication* or safe space* or family support or psychoeducation or CBT).mp. [mp=ti, ab, hw, tc, id, ot, tm, mf, tn, dm, dv, kf, fx, dq, cw, nm, ox, px, rx, an, ui, sy] |
| 295. | (Refugee* or Asylum Seeker* or Survivor* or evacuee* or displace* or IDP).mp. [mp=ti, ab, hw, tc, id, ot, tm, mf, tn, dm, dv, kf, fx, dq, cw, nm, ox, px, rx, an, ui, sy] |
| 296. | 291 and 292 and 293 and 294 and 295 |
| 297. | remove duplicates from 296 |

*Appendix 3: PRISMA Checklist*

| **Section and Topic** | **Item #** | **Checklist item** | **Location where item is reported (page)** |
| --- | --- | --- | --- |
| **TITLE** | | |  |
| Title | 1 | Identify the report as a systematic review. | 1 |
| **ABSTRACT** | | |  |
| Abstract | 2 | See the PRISMA 2020 for Abstracts checklist. | 1 |
| **INTRODUCTION** | | |  |
| Rationale | 3 | Describe the rationale for the review in the context of existing knowledge. | 2-3 |
| Objectives | 4 | Provide an explicit statement of the objective(s) or question(s) the review addresses. | 4 |
| **METHODS** | | |  |
| Eligibility criteria | 5 | Specify the inclusion and exclusion criteria for the review and how studies were grouped for the syntheses. | 4 |
| Information sources | 6 | Specify all databases, registers, websites, organisations, reference lists and other sources searched or consulted to identify studies. Specify the date when each source was last searched or consulted. | 6 |
| Search strategy | 7 | Present the full search strategies for all databases, registers and websites, including any filters and limits used. | 40 |
| Selection process | 8 | Specify the methods used to decide whether a study met the inclusion criteria of the review, including how many reviewers screened each record and each report retrieved, whether they worked independently, and if applicable, details of automation tools used in the process. | 6-7 |
| Data collection process | 9 | Specify the methods used to collect data from reports, including how many reviewers collected data from each report, whether they worked independently, any processes for obtaining or confirming data from study investigators, and if applicable, details of automation tools used in the process. | 6-7 |
| Data items | 10a | List and define all outcomes for which data were sought. Specify whether all results that were compatible with each outcome domain in each study were sought (e.g. for all measures, time points, analyses), and if not, the methods used to decide which results to collect. | 4 |
|  | 10b | List and define all other variables for which data were sought (e.g. participant and intervention characteristics, funding sources). Describe any assumptions made about any missing or unclear information. | 6 |
| Study risk of bias assessment | 11 | Specify the methods used to assess risk of bias in the included studies, including details of the tool(s) used, how many reviewers assessed each study and whether they worked independently, and if applicable, details of automation tools used in the process. | 7 |
| Effect measures | 12 | Specify for each outcome the effect measure(s) (e.g. risk ratio, mean difference) used in the synthesis or presentation of results. | NA |
| Synthesis methods | 13a | Describe the processes used to decide which studies were eligible for each synthesis (e.g. tabulating the study intervention characteristics and comparing against the planned groups for each synthesis (item #5)). | 4 |
|  | 13b | Describe any methods required to prepare the data for presentation or synthesis, such as handling of missing summary statistics, or data conversions. | NA |
|  | 13c | Describe any methods used to tabulate or visually display results of individual studies and syntheses. | 7+9-15 |
|  | 13d | Describe any methods used to synthesize results and provide a rationale for the choice(s). If meta-analysis was performed, describe the model(s), method(s) to identify the presence and extent of statistical heterogeneity, and software package(s) used. | 6-7 |
|  | 13e | Describe any methods used to explore possible causes of heterogeneity among study results (e.g. subgroup analysis, meta-regression). | NA |
|  | 13f | Describe any sensitivity analyses conducted to assess robustness of the synthesized results. | NA |
| Reporting bias assessment | 14 | Describe any methods used to assess risk of bias due to missing results in a synthesis (arising from reporting biases). | 26 |
| Certainty assessment | 15 | Describe any methods used to assess certainty (or confidence) in the body of evidence for an outcome. | 26 |
| **RESULTS** | | |  |
| Study selection | 16a | Describe the results of the search and selection process, from the number of records identified in the search to the number of studies included in the review, ideally using a flow diagram. | 8 |
|  | 16b | Cite studies that might appear to meet the inclusion criteria, but which were excluded, and explain why they were excluded. | 8 |
| Study characteristics | 17 | Cite each included study and present its characteristics. | 9-15 |
| Risk of bias in studies | 18 | Present assessments of risk of bias for each included study. | NA |
| Results of individual studies | 19 | For all outcomes, present, for each study: (a) summary statistics for each group (where appropriate) and (b) an effect estimate and its precision (e.g. confidence/credible interval), ideally using structured tables or plots. | NA |
| Results of syntheses | 20a | For each synthesis, briefly summarise the characteristics and risk of bias among contributing studies. | NA |
|  | 20b | Present results of all statistical syntheses conducted. If meta-analysis was done, present for each the summary estimate and its precision (e.g. confidence/credible interval) and measures of statistical heterogeneity. If comparing groups, describe the direction of the effect. | NA |
|  | 20c | Present results of all investigations of possible causes of heterogeneity among study results. | NA |
|  | 20d | Present results of all sensitivity analyses conducted to assess the robustness of the synthesized results. | NA |
| Reporting biases | 21 | Present assessments of risk of bias due to missing results (arising from reporting biases) for each synthesis assessed. | NA |
| Certainty of evidence | 22 | Present assessments of certainty (or confidence) in the body of evidence for each outcome assessed. | NA |
| **DISCUSSION** | | |  |
| Discussion | 23a | Provide a general interpretation of the results in the context of other evidence. | 22-26 |
|  | 23b | Discuss any limitations of the evidence included in the review. | 26 |
|  | 23c | Discuss any limitations of the review processes used. | 26 |
|  | 23d | Discuss implications of the results for practice, policy, and future research. | 26 |
| **OTHER INFORMATION** | | |  |
| Registration and protocol | 24a | Provide registration information for the review, including register name and registration number, or state that the review was not registered. | 2+4 |
|  | 24b | Indicate where the review protocol can be accessed, or state that a protocol was not prepared. | 2+4 |
|  | 24c | Describe and explain any amendments to information provided at registration or in the protocol. | NA |
| Support | 25 | Describe sources of financial or non-financial support for the review, and the role of the funders or sponsors in the review. | 27 |
| Competing interests | 26 | Declare any competing interests of review authors. | 27 |
| Availability of data, code and other materials | 27 | Report which of the following are publicly available and where they can be found: template data collection forms; data extracted from included studies; data used for all analyses; analytic code; any other materials used in the review. | NA |

*Appendix 4: Mixed Methods Appraisal Tool (MMAT)*

No studies were excluded based on the MMAT however study quality varied. There was a lack of longitudinal design studies and randomised control trials, and sample sizes were often small. Furthermore, some studies incorporated findings into the discussion which did not immediately stem from the research. For example, some studies conducted in China credited the Chinese Government with the efficacy of the MHPSS intervention despite there being no findings of this in the results.


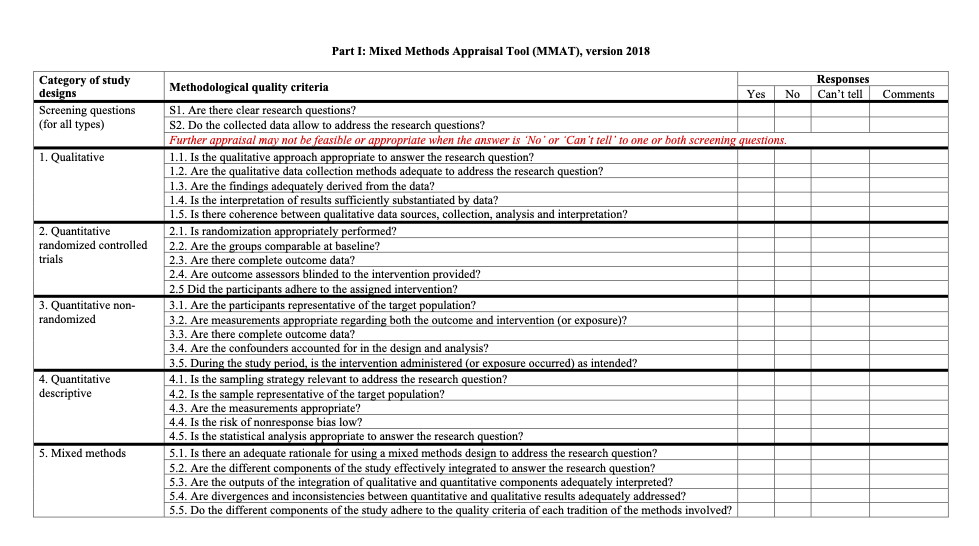

Supplement: Rowe and Nadkarni supplementary material [file S2054425123000912sup001.docx]
